# Supplementary material for: Complex‐centric proteome profiling by SEC‐SWATH‐MS
Source: Mol Syst Biol. 2019 Jan 14;15(1):e8438. doi: 10.15252/msb.20188438 (PMC6346213; doi:10.15252/msb.20188438)
Supplement: Supplementary file 7 — Dataset EV6 [file MSB-15-e8438-s007.zip › feature_plots_bioplex/P55735.pdf]

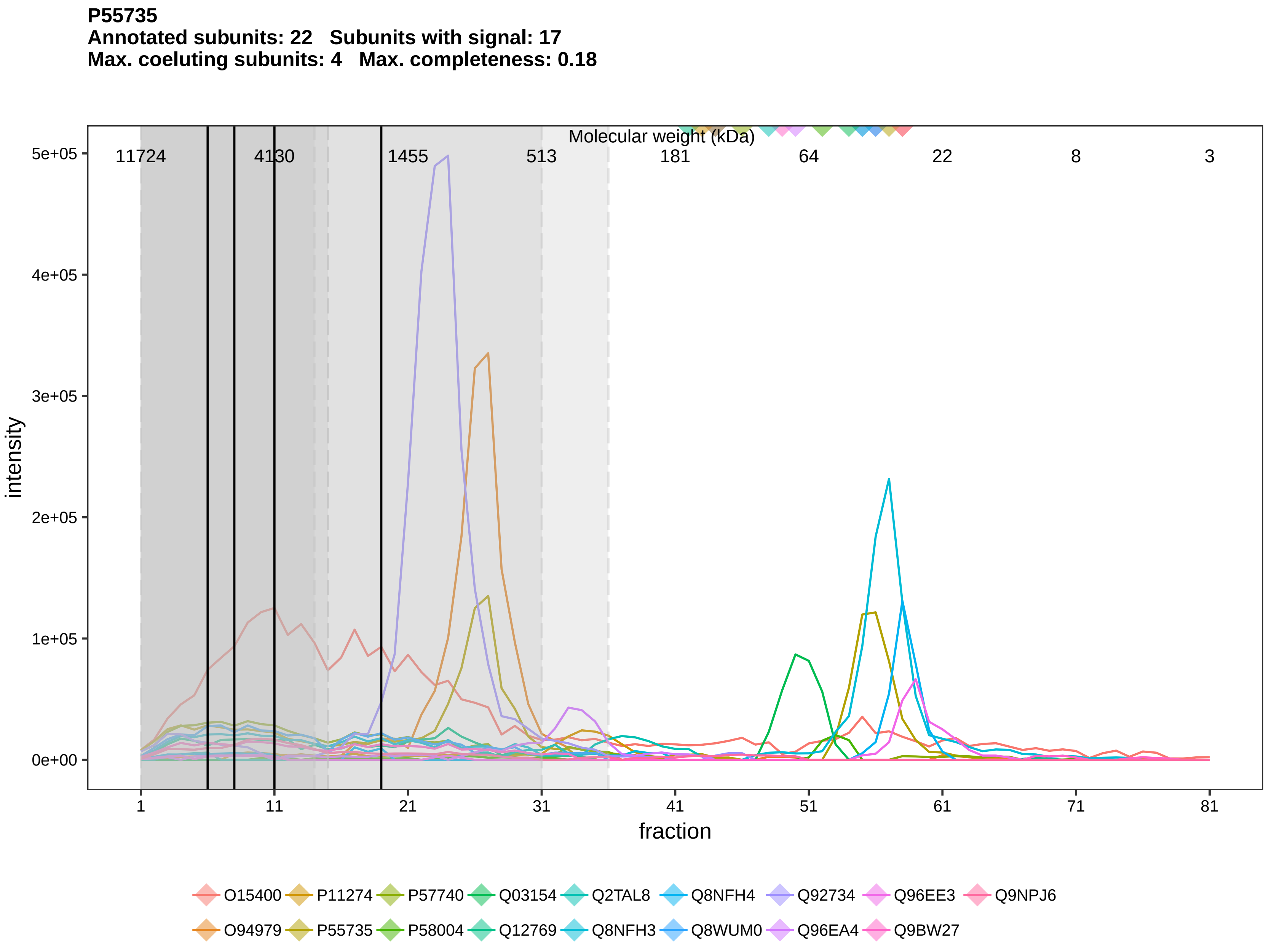
P55735
Annotated subunits: 22 Subunits with signal: 17
Max. coeluting subunits: 4 Max. completeness: 0.18
Molecular weight (kDa)
11724
4130
1455
513
181
64
22
8
3
intensity
5e+05
4e+05
3e+05
2e+05
1e+05
0e+00
fraction
1
11
21
31
41
51
61
71
81
O15400
P11274
P57740
Q03154
Q2TAL8
Q8NFH4
Q92734
Q96EE3
Q9NPJ6
O94979
P55735
P58004
Q12769
Q8NFH3
Q8WUM0
Q96EA4
Q9BW27
